# Variation in ecological scorecards and their potential for wider use

**DOI:** 10.1007/s10661-024-12845-2

**Published:** 2024-07-10

**Authors:** Thomas Gorman, Gesche Kindermann, Kevin Healy, Terry R. Morley

**Affiliations:** 1 Discipline of Geography, and the Ryan Institute, University of Galway, Galway, Ireland; 2 Earth and Life Sciences, School of Natural Sciences, and the Ryan Institute, University of Galway, Galway, Ireland

**Keywords:** Ecological monitoring, Ecological assessment, Rapid habitat assessment, Results-based agri-environmental schemes

## Abstract

Ecological monitoring is a vital tool to help us assess habitat condition and understand the mechanism(s) for habitat change. Yet many countries struggle to meet their monitoring requirements in part due to the high assessment workload. Rapid ecological assessment methods may have an important role to play in this regard. Following their success within several European habitats (e.g., semi-natural grasslands), they are now being developed for additional habitats such as heathlands, peatlands, and other agri-associated areas. Whilst some rapid assessments using ecological scorecards have been shown to be accurate compared to traditional ecological monitoring, less is known about the functionality of these scorecards in heterogenous landscapes. In this study, we selected four existing scorecards to test alongside a prototype. We assessed how these different scorecards measured habitat condition on the same heathland sites. We found that the choice of metrics, their score weighting, and the thresholds used for categorical scores cause scorecards to assess the same site with substantial variation (37%). Vegetation metrics were the primary cause of score variation, with vegetation structure and positive indicator species being the leading causes. Our study indicates that whilst current scorecards may be representative of project-specific goals, they may not be suitable for wider monitoring uses in their current form. Ecological scorecards have great potential to drastically increase the extent of monitoring, but caution is needed before adapting existing scorecards beyond the purposes from which they were designed.

**Supplementary Information:**

The online version contains supplementary material available at 10.1007/s10661-024-12845-2.

## Introduction

**Fig. 1:**
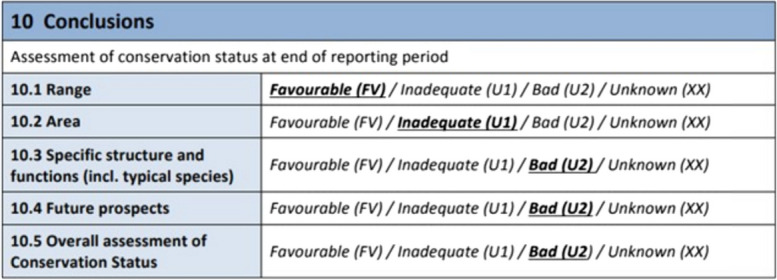
Current ecological condition assessment of dry heath (4030) habitat in Ireland (NPWS, 2019b)

The most recent assessment on the status and trend of European habitats concluded that only 15% of relevant protected habitats have sufficient area and quality (i.e., are in *favourable*^1^ condition; EEA, 2020). In the European Union, habitat protection is afforded through two nature directives, the Habitats Directive and the Birds Directive (92/43/EEC, 2009/147/EC respectively). They aim to ensure the conservation of a wide range of rare, threatened, or endemic animal and plant species as well as characteristic habitat types. The Habitats Directive, adopted in 1992, notably aims to maintain or restore habitats to ‘favourable’ condition and now represents through its Natura 2000 network of protected sites, the largest coordinated network of nature conservation areas in the world. An example of the current habitat assessment criteria is shown in Fig. 1.

Successful implementation and evaluation of conservation efforts such as those required under the Habitats Directive require regular monitoring of habitat quality. Ideally, these assessments are dependent on detailed condition and status assessments, requiring in-depth ecological survey methodologies, which are to be repeated every six years as part of the Article 17 reporting requirements for every Member State. However, this is rarely accomplished for all protected areas on a national scale. There is a paucity of comprehensive data on many habitats and species, with a majority of the condition assessments comprised of partial survey and expert opinion (EEA, 2020). Ireland is no exception. The most recent assessment of the condition of Irish (Annex I) habitats states that only approximately 15% are in *favourable* condition, with only 2% having their condition trend ranked as *improving,* meaning that the habitat is expected to be in better condition at the time of the next round of ecological monitoring. Furthermore, approximately 70% of the data used to establish condition trends are extrapolated from other data or based upon expert opinion (NPWS, 2019a). If we lack the means to increase our monitoring capabilities, there is an imperative for cost-effective monitoring systems (Balmford et al., 2003; Haase et al., 2018; Vos et al., 2000).

Ecological monitoring serves as a crucial tool for tracking and preventing biodiversity loss (Lovett et al., 2007), yet in the European Union, Member States struggle to unify methodologies and to adequately meet their monitoring requirements (Delbosc et al., 2021; Ellwanger et al., 2018; Vanden Borre et al., 2011). Financial resources are often the limiting factor in the scope of monitoring programs; therefore, the design choices of what is monitored, how often, and where, are critical to justifying the costs (Caughlan & Oakley, 2001). Rapid ecological assessment methods have become paramount to address these monitoring issues (Díaz-Delgado et al., 2017).

Rapid habitat assessments provide a cost-effective measure of the ecological status of habitats using a set of indicators that can be reliably assessed during a single field visit. They are not intended as a replacement of in-depth ecological surveys, but rather to provide a tool to increase the scale of monitoring by reducing both the level of expertise required and the time needed to survey (Medeiros & Torezan, 2013). These methods often produce a ‘site score’, where ecological condition is represented on a numerical scale. These were typically designed to provide guidance for biodiversity off-setting decisions, where in-depth biological survey data is not necessarily the primary focus. Biodiversity offsetting has been developed as a strategy to balance the needs of conservation and development, which relies on measurable conservation efforts and the principle of no net loss (Bull et al., 2013). Examples of these type of systems can be found in Australia (e.g., ‘Habitat Hectares’ (Parkes et al., 2003), the USA (e.g., ‘Habitat Suitability Indices’ and ‘Habitat Evaluation Procedures’ USFWS, 1981, (Brooks, 1997)), and Europe (Elmiger et al., 2023). Several rapid assessment approaches use ecological scorecards, such as those developed for recent European agri-environmental schemes (AES).

In an attempt to alleviate the negative impacts of food production on biodiversity, AES have been developed throughout Europe since the 1990s and have become mandatory for Member States since the reforms to the Common Agricultural Policy (CAP) in 1999 (Uthes & Matzdorf, 2013). The aim of these schemes is to reduce farming pressure on ecosystems and to protect natural and semi-natural features associated with farmed landscapes by compensating farmers for using less intensive agricultural practices, incentivising for increased biodiversity, or to subsidise farming in areas that are no longer economically viable (Batáry et al., 2015; Hodge et al., 2015). On the surface, AES seem an ideal solution. However, the effectiveness of AES has been questioned and ecological improvements have been hard to measure (Concepción et al., 2008; Kleijn & Sutherland, 2003; Pe’er et al., 2021).

In response, targeted, often results-based AES that engage farmers in the decision-making process have been a successful solution to traditional AES. They relay ecological management advice and measure effectiveness via a purpose-built ecological scorecard and are now seen as a major component of AES (Moran et al., 2021; Rotchés-Ribalta et al., 2021). The advantage of the numerical score in these systems is to provide a scalable financial reward for biodiversity improvements, rather than to offset potential biodiversity losses. The rapid assessment allows for single-site surveys to be repeated yearly and rewards ongoing improvements in the site score. Following the successes of projects employing this method, such as BurrenLIFE (Burren Programme – Farming for Conservation, 2021) and Results-Based Agri-environment Payment Schemes (RBAPS Project, 2021) across Europe, these results-based AES have seen widespread adoption through the Agricultural European Innovation Partnerships (EIP-AGRI), particularly in Ireland. The ecological scorecards developed in these programs have been primarily used to assess a variety of semi-natural grasslands (e.g., managed grasslands with reduced grazing regimes and fertilizer application), but recent efforts have been made to incorporate additional habitats that also fall within the agri-environment. Successful examples of these include heathlands to protect Hen Harriers, and farming practices near waterbodies to protect endangered mussels (e.g., Hen Harrier Project 2021; Pearl Mussel Project, 2021). The scorecards in these projects were developed from a draft peatland card proposed as part of the RBAPS project and are designed to cover blanket bog, dry and wet heath, and mosaics of heathland and grassland (Sullivan & Moran, 2017).

The reduction in the need for expert knowledge and the rapid assessment design of ecological scorecards could place a much needed and powerful habitat monitoring tool into the hands of a wider demographic. The allure of this should not be underestimated. Project-focused ecological scorecards are seeing a substantial increase in recent years and have now been incorporated into the recently rolled-out Agri-Climate Rural Environment Scheme in Ireland (ACRES, 2022). Whilst grassland scorecards have been extensively tested against traditional biological sampling (RBAPS Project, 2021), it is important to understand if the selection of scorecards for additional habitats is suitable to accurately reflect ecological condition. As peatland restoration becomes a policy priority in both Ireland and the EU (CAP23, 2023), we feel it is timely to assess the available rapid assessment methods used in recent AES to establish if there is variation in the results. In this paper, we aim to establish the level of variation between five ecological scorecards designed to assess heathland and peatlands. We attempt to answer whether the choice of card will alter the site score, or do all scorecards produce a similar measure of ecological condition. We carry out a case study scoring the same sites with five scorecards simultaneously, followed by score simulations in R (R Core Team, 2023). We compare the overall site scores and then assess the score contributed by each ecological indicator within each scorecard to identify the driving forces of the variation.

We use the score simulations to expand the sample size by replicating potential site scores for each card to determine if the card design influences site scores independent of site ecological condition. We then compare it to our findings in the field.

## Methods

**Fig. 2:**
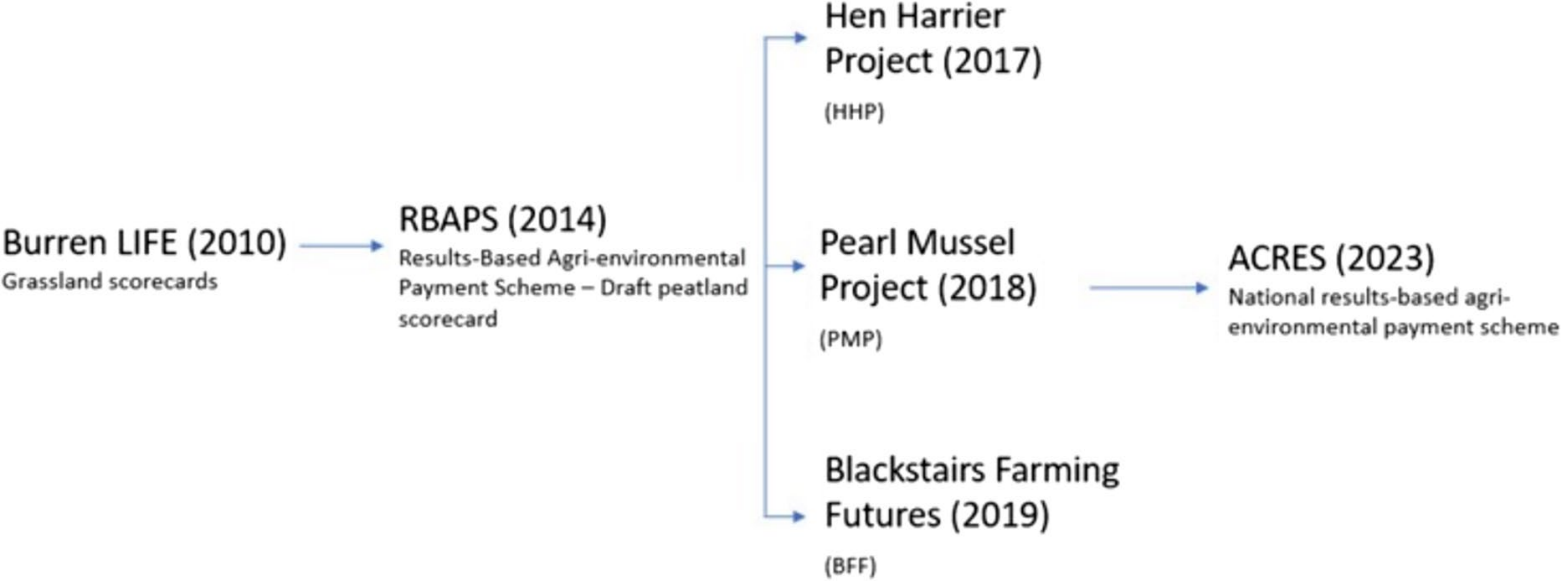
Progression of results-based agri-environmental payment schemes using ecological scorecards. The Test card was developed in 2017 to assess machair habitats, and the methodology was adapted upland habitats in 2019

We tested the four existing peatland and heathland scorecards that were designed by others for use in different AES projects in Ireland to test alongside a new prototype developed from a scorecard framework that includes landscape metrics (herein referred to as Test) (Gorman et al., 2019). The existing cards all have a common origin in the cards developed from the BurrenLIFE project and RBAPS project. These were chosen from the Blackstairs Farming Futures (BFF) project, the Hen Harrier Project (HHP), the Pearl Mussel project (PMP), and the draft peatland scorecard from the RBAPS project, which was created as an example of how peatland/heathland scorecards could work. The Test scorecard was developed on similar principles but used monitoring stops rather than a single walkover and could incorporate the surrounding environment, which is outside of the scope of current scorecards. These scorecards also influenced the design of the scorecards used in ACRES national agri-environment scheme, created after this study was completed (Fig. 2).

### Site selection

**Fig. 3:**
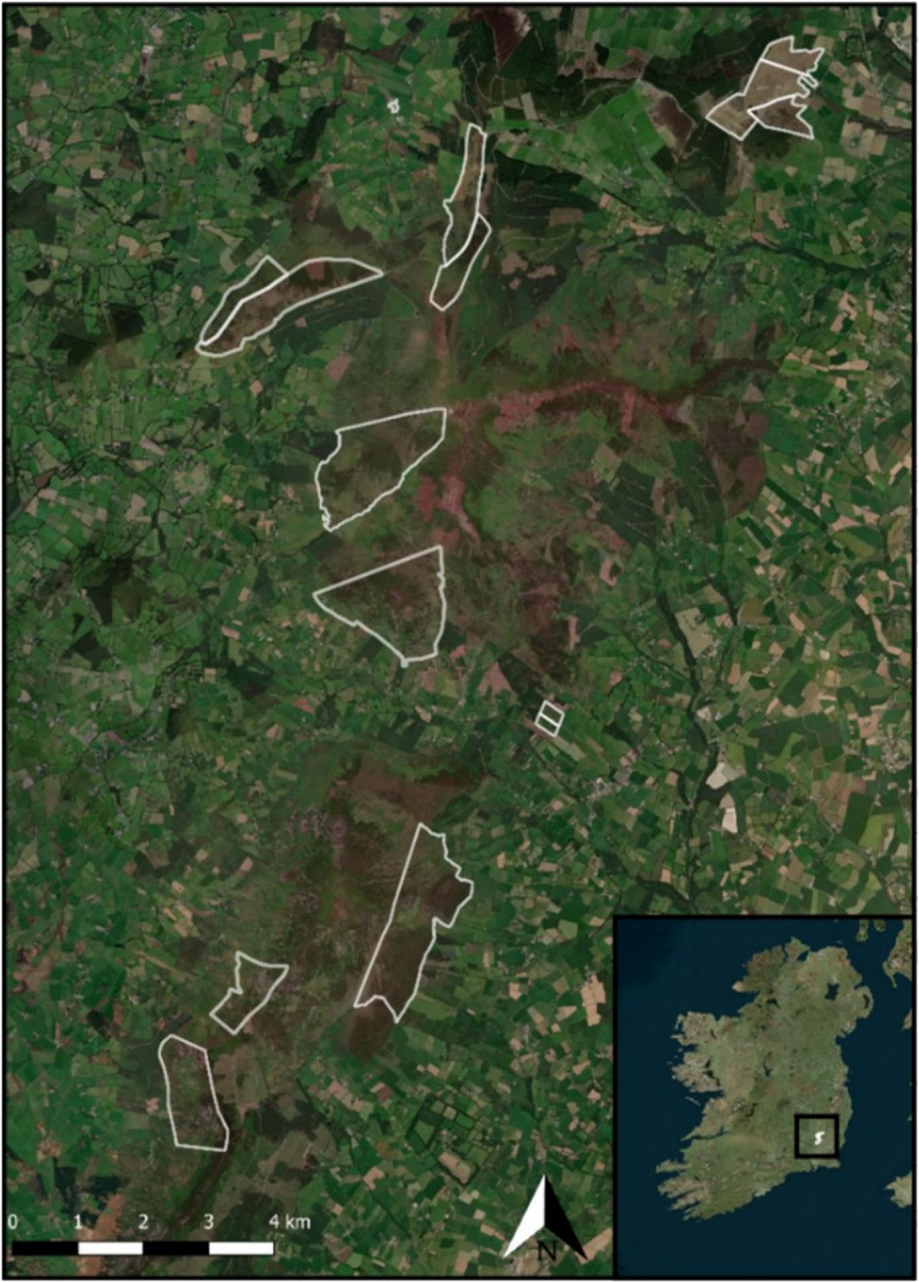
Selected field sites in the Blackstairs Mountains, Co. Carlow & Wexford, Ireland. Field sites are outlined in white, based on commonage boundary records. Aerial imagery: Bing Maps, 2022

To test the scorecards on the range of habitats they are designed to cover, we focused on the Blackstairs Mountains Special Area of Conservation (SAC) (Fig. 3). Under the Habitats Directive, this area is protected for three Annex I habitats, which are European dry heaths [Annex code: 4030] and Northern Atlantic wet heaths with *Erica tetralix* [Annex code: 4010] which often occur as patchy habitat mosaics. There are also areas of upland blanket bog [Annex code: 7130] and a mixture of grassland habitats, which are not listed on Annex I of the Habitats Directive. This provided a good match for the habitats covered in the existing peatland and heathland scorecards. Nine sites were selected as part of the Blackstairs Farming Futures EIP, and we used these project sites for our analysis. The site names have been changed to numbers to protect sensitive landowner information.

The heathlands of the Blackstairs Mountains are deemed to be in *favourable* condition overall, although some areas are damaged due to inappropriate fire management and grazing regimes (Tubridy et al., 2015). Inappropriate stocking levels and the overuse of fire management have led to increasing bracken cover in many places as well as exacerbated erosion rates on the upper slopes.

### Site survey methods

**Fig. 4:**
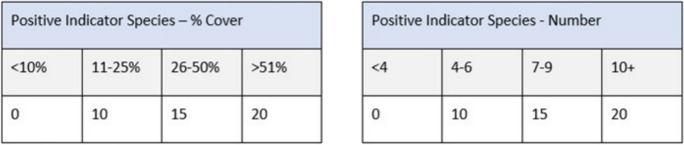
Positive indicator species proxy indicators used in the Blackstairs Farming Futures heathland scorecard. The surveyor uses a site-wide visual assessment to select the appropriate categorical score, based upon the defined threshold

All five scorecards were scored simultaneously during a single site walkover following a structured walk (where possible) to cover a representative sample of the scoring area. Typically, a ‘structured W’ shape is used, where an assessor walks a path in the shape of a W to observe as much of a representative area as possible in a single visit. However, this can be impractical to carry out in upland habitats due to difficult or dangerous terrain. Therefore, before each site visit, we planned a route that would cover the lower, middle, and upper slopes throughout the site. Deviations could be made enroute where the terrain became impassable. We assessed a series of proxy indicators (e.g., Fig. 4) of habitat condition used by the selected scorecards either during the walk or upon completion of the walk. The test scorecard follows the methodology set out in Gorman et al. (2019).

**Fig. 5:**
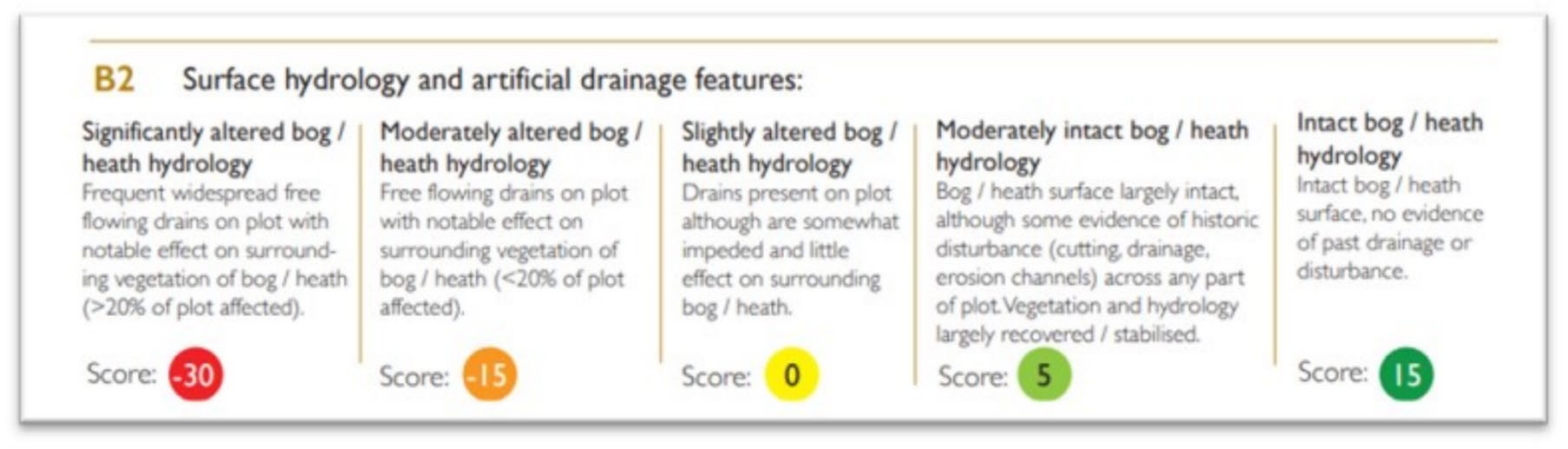
Score categories for the impact of drainage on a survey site, taken from the Pearl Mussel Project peatland scorecard (Pearl Mussel Project, 2021)

These categorical scores can carry a positive or negative score and some variables may contain a mixture of both, such as the example in Fig. 5.

### Defining assessment area

The scorecards included have been developed for the purposes of encouraging ecologically beneficial agricultural practices and so they are primarily designed to score contained field sites, rather than the full habitat extent. This establishes clear site boundaries based on land ownership even if physical boundaries do not exist, as may be the case with upland unenclosed grazing practices such as commonage.

### Ecological indicators

Sites were scored using a suite of proxy indicators of ecological condition based upon three main parameters: vegetation, hydrology, and site management. These indicators rely on visual assessment (e.g., percent cover) of the site on a single visit. Vegetation indicators comprise of the number of pre-defined positive and negative indicator species found throughout the site, where positive indicators are those species affiliated with the habitat and negative indicator species (e.g. *Pteridium aquilinum, Juncus effusus*) are reflective of either poor condition or invasive species. The indicator species vary for each scorecard, but positive indicator species typically include *Calluna vulgaris, Vaccinium myrtillus*, and *Galium saxatile*, whereas negative species would include plants that indicate enrichment, such as *Urtica dioica*, or forestry plantation escapees such as *Picea sitchensis*. Often, cryptic or difficult to identify species are grouped into an aggregate, such as brambles (*Rubus* spp.) or sedges (*Carex* spp.). Non-species-specific indicators such as the percentage cover of bryophytes and lichens, and the overall structure of vegetation within the site boundaries, are included in some of the scorecards, though these often rely on the experience of the assessor and require calibration to reduce user error (Morrison, 2016).

Hydrological indicators are based on the visual assessment of features such as artificial drainage and impacts upon natural watercourses within the site. The site management indicators are also visually assessed by a surveyor throughout the site visit. These can include damage caused by burning, supplementary feeders, or resource extraction.

### Variations in scorecard design

Scorecards mostly use a similar set of metrics (Table 1), though the weightings and thresholds for them vary to reflect individual project goals. Whilst the chosen scorecards all have a common origin and design, there are some key differences. For instance, the BFF and RBAPS cards both assess grazing levels, whereas the others assess the impacts of grazing through vegetation structure and supplementary feeding. Another notable difference is that the HHP card does not assess positive indicator species.

**Table 1:** Description of habitat condition proxy indicators typically used in Irish results-based scorecard systems, sorted by general category. Indicator thresholds for each scorecard are also listed as scorecards may use the same indicator but set different parameters for scoring. The absence of metric is indicated by a dash (-). Scorecards assessed are from the Blackstairs Farming Futures project (BFF), the Hen Harrier Project (HHP), the Pearly Mussel Project (PMP), Results-Based Agri-environmental Payment Schemes (RPABS), and a newly designed prototype (TEST)

Indicator	Description	BFF	HHP	PMP	RBAPS	Test
**Vegetation indicators**						
**Positive indicator species – number**	Site-wide presence/absence	10 species from 22	−	9 species from 16	9 species from 13	9 species from 22
**Positive indicator species – cover**	Site-wide visual estimate	> 50% cover	−	−	> 50% cover	−
**Negative indicator Species – number**	Site-wide presence/absence	−	Invasives (any)	Invasives (any)	Negative indicators and invasives (any)	−
**Negative indicator species – cover**	Site-wide visual estimate	< 5% of site	< 5% of site	Absent/ < 1%	Absent	−
**Vegetation structure**	Site-wide visual assessment	Expert opinion	Expert opinion	Expert opinion	−	Expert opinion
**Bracken cover**	Site-wide visual estimate	< 10% to > 50%	< 10% to > 50%	−	−	−
**Scrub cover**	Site-wide visual estimate	< 10% to > 50%	Absent/small areas of natural scrub	−	−	< 5% to > 50%
**Bryophyte cover**	Site-wide visual estimate	< 10% to > 25%	−	< 5% to > 30%	−	< 10% to 25%
**Hydrological indicators**						
**Drainage**	Site-wide visual assessment	Active drains	Active drains	Active drains	Active drains	Active drains
**Impact on watercourse**	Visual assessment of watercourses	−	Livestock access	−	−	−
**Contribution to watercourses**	Visual assessment of watercourses	−	−	Presence/absence of wet features	−	−
**Site management indicators**						
**Grazing**	Site-wide visual estimate	Expert opinion	−	−	Expert opinion	−
**Bare soil**	Site-wide visual estimate	> 5%	> 5%	> 10%	> 5%	> 5%
**Burning**	Site-wide visual assessment	None/controlled	None required or evident	No evidence	None required or evident	None/controlled
**Turbary (peat extraction)**	Site-wide visual assessment	−	None, or < 5% of site hand cut	No cutting for > 2 years	−	−
**Supplementary feeding**	Site-wide visual assessment	−	No damage	No damage	Low	−
**Damaging activities**	Site-wide visual assessment	None/low	None	None	−	None

For the ease of communication with landowners, scorecards have a set maximum score of 100 points, which is presented as a score out of ten. The test scorecard is being developed free from the restrictions of results-based schemes, allowing it to incorporate the broader landscape, and uses five monitoring stops rather than a single walk over. The Test scorecard gives a score out of 500, which has been turned to a percentage to compare against the other scorecards.

Guidance is provided for indicators that are based on expert opinion, such as vegetation structure. To score positively in this indicator, the user is instructed to assess variation in vegetation height and species composition, with a mixture of open areas of grass and sedges, patches of mixed aged heather and shrubs, and well-defined bryophyte layer scoring highly. Alternatively, sites with uniform vegetation height and communities are scored poorly, as they often reflect over or under grazing.

There are some differences between the positive indicator species lists used for each card. The BFF scorecard uses the most species (22) and the PMP uses the least (16). The difference lies primarily in the shrub layer, with the BFF card scoring *Vaccinium myrtillus, Empetrum nigrum*, *Arctostaphylos uva-ursi*, and *Vaccinium vitis-idaea*, whereas the PMP only scores for *Vaccinium myrtillus*. The BFF card also counts emergent scrub species such as *Corylus avellana, Prunus spinosa*, and *Rubus* spp*.* Provided the total cover is less than 10%. Over this threshold, they become negative indicators. The PMP only recognises them as negative indicators, scoring them negatively at over 1% cover.

### Scorecard comparison

To establish the extent of variation between scorecards, we first compare the overall site scores achieved by each scorecard. The individual scores for each site are compared, as are the average scores achieved by each card. To address the small sample size, we use score simulations to determine if scorecard design is likely to alter the condition score and if this is reflected by our findings in the field.

We then analyse the percentage score contributions for each of the indicators to establish the drivers of score variation in each card.

### Scenario testing

We tested the potential for scorecard variation by creating a simulation program of the scorecards under three scenarios. First, we ran the scenarios under a random score generator, where each categorical score was equally likely to be chosen. As this is unlikely to be the case in the field, we next ran a scenario with ‘biased’ and ‘correlated’ scores. Biased scores were based on our findings in the field by weighting the probability of a category being selected. For example, burning and bracken cover were both common negative scores in the field sites, so we increased the probability of a negative score. Correlated scores were based on the likely influence of other indicators, such as a site that scored poorly for burning and bare soil would be unlikely to have good vegetation structure. We replicated each scenario 1 million times using the R programming language (R Core Team 2021). The chosen model will be used to replicate site scores to establish whether the choice of scorecard is likely to alter the resulting score independent of ecological condition in the field. The corresponding R code is supplied in the Supplementary Material.

## Results

Using the five selected rapid assessment approaches, we scored nine upland heaths to explore the potential variation between the different scorecards used. Because of the variation inherent in each scorecard, the scores were standardised to provide a score out of ten. Overall, we found variation in the selected scorecards to be 37%, with the leading factors being vegetation structure and positive indicator species (31% and 19%, respectively). The maximum score contributions for each indicator are shown in Table 2, where we have grouped them into their relevant categories of vegetation, hydrology, and site management. The number of indicators used, and the potential points lost is not standardised between cards. The scores produced by each scorecard varied by an average of 3.7 points (out of 10) across nine sites. On individual sites, scores varied by a minimum of 1.5 points (sites 2 and 9) and a maximum of 6.4 (site 8). Site rankings were inconsistent. Site 2 scored highly (7 or above) on each scorecard, site 6 scored poorly (2.3 or less), and site 9 scored between 6 and 7.5, but the remaining six sites were inconsistent across the five scorecards (Appendix). The RBAPS draft scorecard’s average was the lowest (3.6), whereas the Test scorecard gave the highest overall scores (6.7).

**Table 2:** Analysis of the scoring systems of the five selected scorecards, showing maximum score contributions for general indicator categories and the number of indicators used. The potential for points loss is the total of negative scores that can be given by proxy indicators per scorecard. The average score (out of a maximum of ten) is taken from the scoring of nine sites

Scorecard	Vegetation (%)	Hydrology (%)	Site management (%)	Number of indicators	Potential point loss	Average score (-/10)
BFF	70	15	15	12	125	5.8
HHP	60	20	20	12	190	4.4
PMP	55	30	15	12	180	5.0
RBAPS	50	20	30	10	120	3.6
Test	54	12	34	10	565	6.7

### Score contributions

**Fig. 6:**
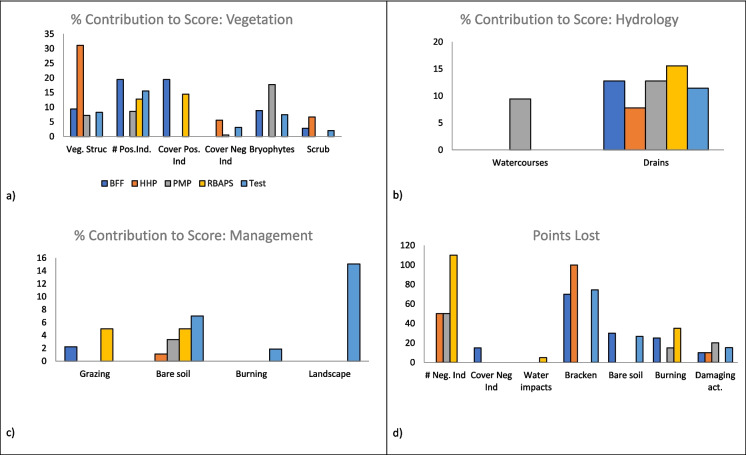
Percentage contribution of each proxy indicator to total scores achieved. The percentage for each indicator is calculated against its potential maximum contribution. Indicators are grouped by general category with zero scores omitted

To establish the drivers of score variation, we assessed the total score of individual variables across all sites. Vegetation structure provided the most points across all scorecards and bracken cover was the leading cause of points loss. The percentage contribution to the total scores for each indicator are shown in Fig. 6.

The number of negative indicator species in the RBAPS card is the largest source of points lost and one the key factors in the RBAPS card having the lowest average score (Fig. 6d). Threshold differences for the number and cover of negative indicator species and bare soil was also a contributing factor. The number and cover of positive indicator species were the main contributors to the higher score achieved in the BFF card. The Test card scored higher for drainage and positive indicator species number.

### Metric Variation

**Fig. 7:**
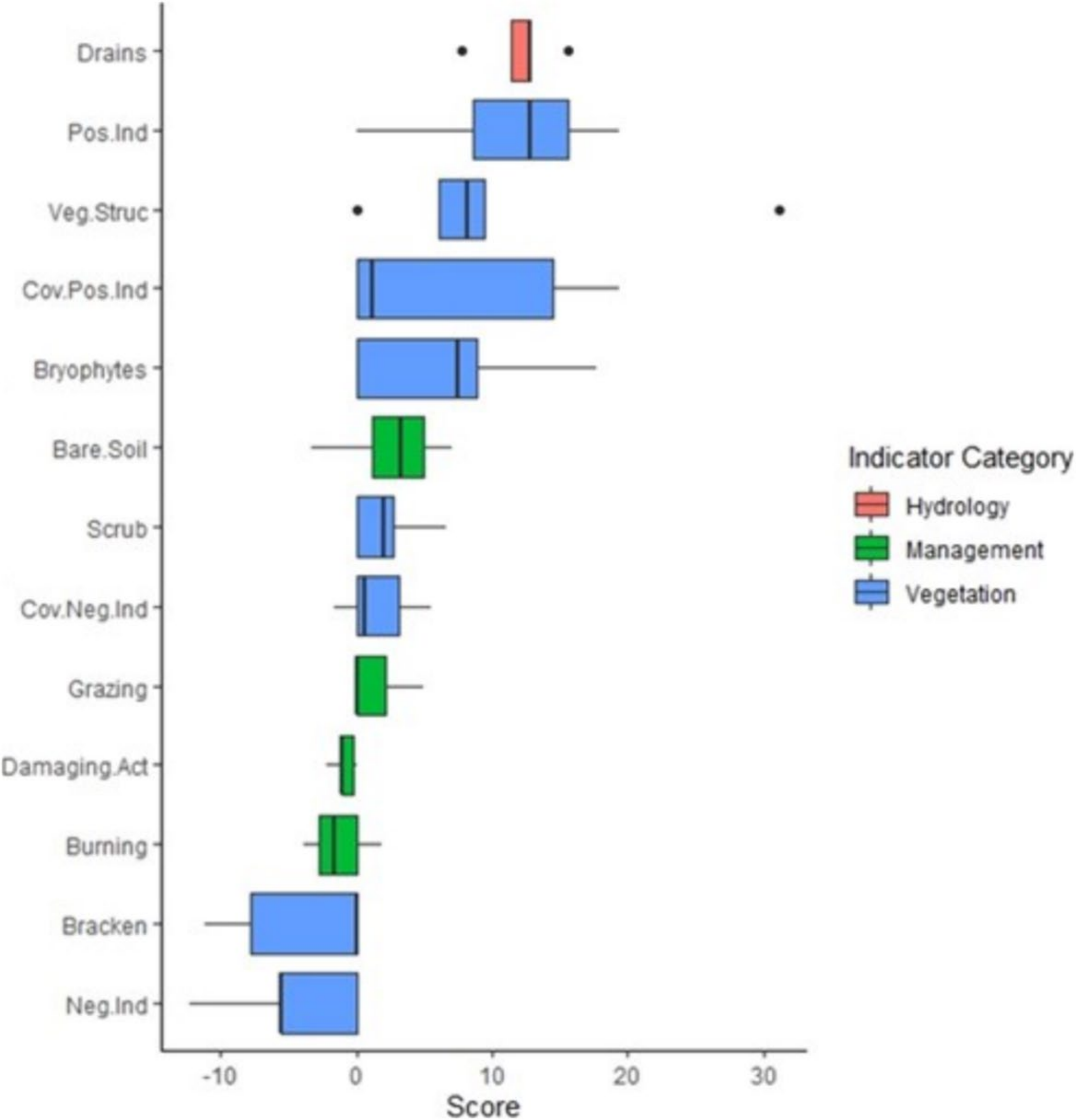
Score contribution variation among proxy indicators used in ecological scorecards, sorted by median score. Total score contributions for each indicator are combined and compared between scorecards. Vegetation-based indicators typically have the greater variation. Zero scores omitted

The main sources of variation are the vegetation metrics, with the number and cover of positive indicators, and vegetation structure having the broadest range of contributions across cards. Vegetation structure has the highest level of variation (6.1% to 31.1%) whereas damaging activities had the lowest variation (1.1% to 2.2%) (Fig. 7).

### Scenario testing

**Fig. 8:**
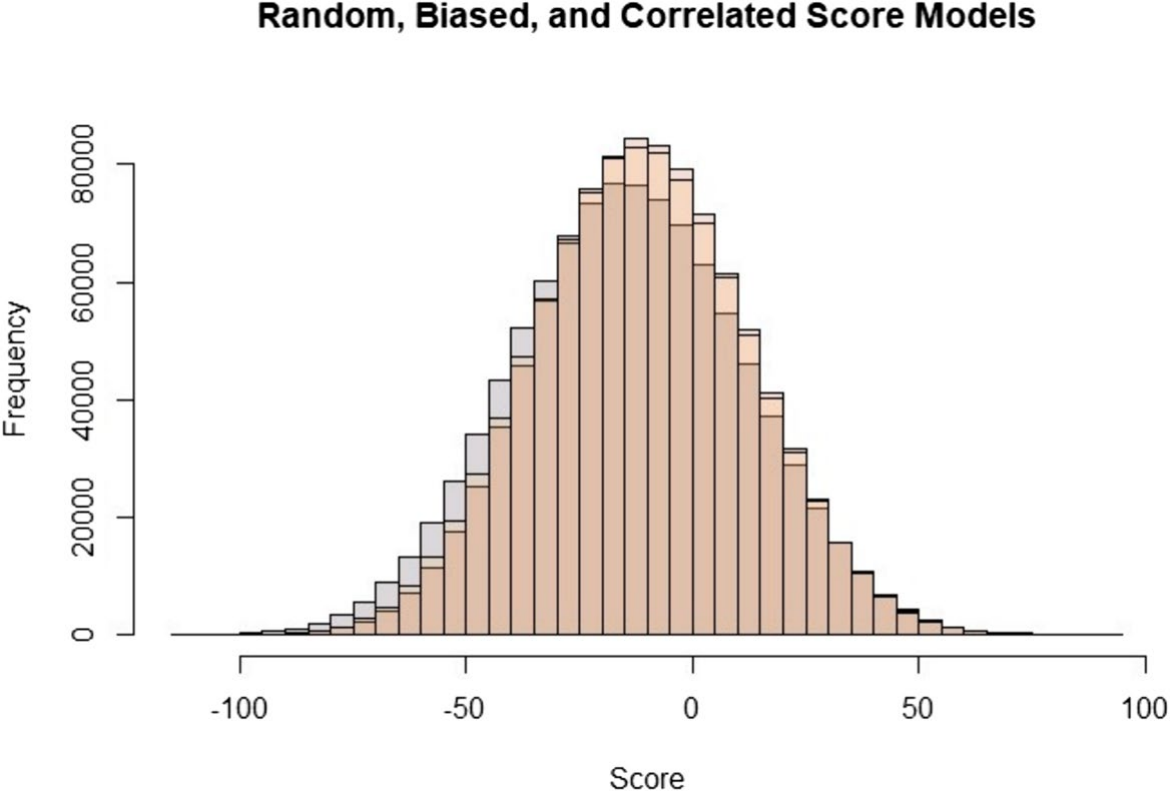
Histogram displaying the results of scorecard simulations using random, biased, and correlated models. Mean scores are − 11.55, − 8.26, and − 8.95, respectively

Using the three scoring simulations (random, biased, and correlated), we ran 1 M simulations of each and found the mean to be biased negatively, at − 11.55, − 8.26, and − 8.95, respectively (Fig. 8).

We found no significant difference using a one-way ANOVA between the random and the biased simulations (*F* = 0.0009, *P* > 0.05), and the random and correlated simulations (*F* = 0.447, *P* > 0.05).

**Fig. 9:**
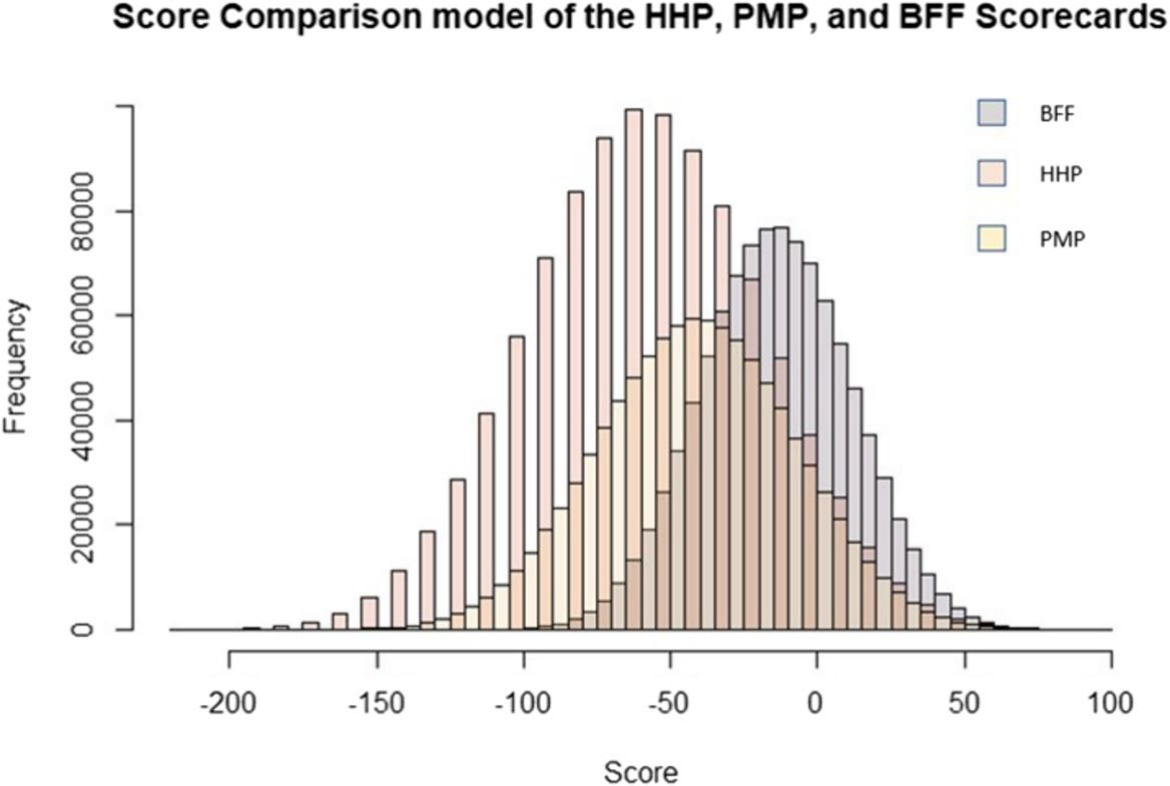
Random score model for the three peatland scorecards used in the Blackstairs Farming Futures project (BFF), the Hen Harrier Project (HHP), and the Pearl Mussel Project (PMP)

We then applied the random simulation to three of the selected scorecards. We chose the scorecards which are currently in use, omitting the draft (RBAPS) and prototype (Test) cards. The simulations matched our findings in the field, with the BFF card scoring sites higher on average (mean =  − 11.57) and the HHP scoring lowest (mean =  − 56.57), with the PMP card scoring between them (mean =  − 39.54) (Fig. 9).

Carrying out an ANOVA between the three scorecards showed a significant difference in the ecological score, depending on the scorecard chosen (*F* = 472762, *P* > 0.05).

## Discussion

We compared the results of five heathland and peatland scorecards in the Blackstairs Mountains SAC to understand how scorecards assess ecological condition on the same site. Site scores rarely matched, with a minimum difference of 1.5 points and a maximum of 6.3 out of 10. Scorecards may be reflective of project-specific goals but may not be suitable for wider monitoring in their current form. However, with some adaptations, the methodology could become a valuable tool outside of results-based AES.

### Scorecard comparison

The selected scorecards most often use 12 indicators out of 18, with only drainage, burning, and bare soil being used by all scorecards. Grazing (two cards) and secondary indicators of hydrological condition (one card each) are the least used indicators. The RBAPS draft card scored sites the lowest on average, whilst the Test card scored sites most favourably. Of the scorecards currently in use, the HHP scored sites lower than both the PMP and BFF. Due to the small number of field sites in this case study, we decided to use score simulations to replicate potential site scores using random score distributions. Applying a random score generator to each scorecard produced similar results to our field data, suggesting that the choice of scorecard will impact the site score due to card design rather than variation in ecological condition.

Despite assessing similar metrics, there are some key differences between scorecards. Whilst vegetation-based indicators typically provide the largest contribution (50–70%) to the total score, substantial differences occur within this category, and it is the primary cause of score variation. This is likely a result of project-specific goals. For example, the largest scorecard deviation (HHP) does not assess either positive indicator species number or cover, but instead rewards a potential 40% of the total score for vegetation structure. The PMP places more weight on bryophyte cover and hydrology than species present, whereas the BFF card places greater emphasis on positive indicators with 40% of the total score available for number and cover (20% each). The weightings of these indicators make sense for the specific project; however, the data is of limited use for assessing underlying habitat condition if used outside of these aims.

The score weighting is not solely responsible for score variation, as the thresholds and qualifiers set for potential points loss can also have an effect. For instance, the RBAPS draft card penalises for the number of negative indicator species and invasive species together, meaning that the detection of one individual from the list of negative indicator species can reduce the score and this is the leading cause for this card scoring sites lowest on average. This variable is either assessed by percentage cover or by separating negative indicators and invasives in the other scorecards. Threshold testing has been rarely carried out in European ecological score indexes to date (Elmiger et al., 2023). As our data shows, this can lead to variation in the ecological score, which has both financial implications for landowners in results-based AES and obscures the overall ecological condition of the site. Testing and calibration requires experts in the field, and so can add to project expenses. However, scenario testing and modelling may help limit this. Despite the merits, modelling has yet to find a regular role in the quantitative estimation of ecological condition, such as scorecard design and biodiversity offsetting (Borges-Matos et al., 2023). A relatively simple program such as the one used in our study could help with the creation of new scorecards by testing it against a benchmark, set by a previously tested scorecard. This would allow creators to test if their scorecard is likely to score sites above or below the benchmark, saving valuable field time.

Variation was low overall in site management indicators. This was due to the similarity in the thresholds and descriptions across most scorecards. A caveat to this is that none of the sites surveyed are subject to turbary (peat extraction) and supplementary feeding, whilst damaging activities are infrequent. However, burning and bare soil are common in this region and the thresholds differ between cards. The key difference for burning is that the BFF and Test scorecards score low burning evidence as neutral, whereas the other cards only score neutral or positive for no evidence of burning. Similarly, the BFF, HHP, and RBAPS card sets the threshold for the lowest category score (− 2 and − 1.5 respectively) for bare soil cover at > 5%, whereas the PMP sets it at > 10% (− 2). Hydrological indicators are mostly limited to drainage and similar guidance is used throughout the selected scorecards. The variance in this category is due to the PMPs stronger weighting, reflecting the projects focus on water quality.

Scorecards typically have a maximum score of 100, but the potential points that can be lost is not standardised. When a metric does not score maximum in its category, the overall achievable score is reduced (i.e., if a metric can score 20 points and scores 0, then the maximum score is now 80). This is the cause of the negative means in the random score simulations. However, this data matches the infield findings and suggests that the lack of standardised points loss across cards does influence the average score achieved by each scorecard. Adding a negative score compounds the effect. An example of this is that the PMP and HHP both scored a site below zero (Appendix). An indicator that can score negatively and positively has an increased weighting, as it now can score across a wider range. For example, the PMP scorecard scores drainage from a range of + 15 to − 30. This gives a combined weighting of 45 points, which is 45% of the potential score on one indicator. The overweighting of indicators can lead to ‘attribute eclipsing’, where one indicator overshadows the rest. This problem was identified in three Australian rapid assessment methodologies for biodiversity offsetting (Oliver et al., 2014). Another problem these methods can face is the combination of positive and negative scores leading to all sites scoring average, despite clear differences in condition. This could potentially be the case with the BFF scorecard, as most sites scored maximum points on the positive indicator species metrics. A review of wetland rapid assessment methods in the USA showed that two systems tested on the same sites produced ‘medium’ ranked scores persistently, despite the field sites showing apparent differences in condition (Fennessy et al., 2007).

A survey of ecologists currently using scorecards feel that they are a ‘fairly accurate’ representation of ecological condition (Gorman, unpublished data); however, such variance suggests that they may be accurate to project goals but not necessarily true condition. The question that must be asked regarding scorecard methodologies is what are we measuring? The purpose the card is created for must be considered before any assumptions are made as to whether it represents true ecological condition. Ecological scorecards have proven to be a valuable tool in results-based systems, but in their current form, they are unlikely to be suitable for wider uses.

### Recommendations for developing scorecards outside of results-based AES

One of the many benefits to ecological scorecards is that they are easily adaptable to new locations or purposes; however, we found that caution is needed before assuming an existing card will be appropriate for new project objectives or habitats. Designing a new card should not be ruled out through the assumption an existing one will be adequate, as the choice and weighting of indicators may need to be adjusted.

The use of positive indicator species can be problematic for scorecard design. For a single survey-based methodology, plants are the only viable taxa, but the number and choice of indicator species deserve particular attention as their detection and correct identification can be a major source of error, especially in simple presence/absence surveys (Elmiger et al., 2023; Milberg et al., 2008; Scott & Hallam, 2003). Furthermore, heterogenous landscapes such as the uplands can have incompatible species lists. Positive indicator species lists for specific habitats are detailed in established monitoring methodologies (JNCC, 2009; Perrin et al., 2014); however, scorecards that cover multiple habitats make the selection of species either too large to be practical or too vague to be accurate. Despite concerns regarding the suitability of indicator species to reflect broader trends (Lindenmayer & Likens, 2011), they are still gaining popularity as a cost-effective tool to measure a variety of ecological issues (Siddig et al., 2016).

Proxy indicators can be vulnerable to sampling bias, observer bias, and measurement errors as shown in an evaluation of the US ecological integrity assessment (Brown & Williams, 2016). Further problems may arise with the biases inherent in converting raw data into categorical scores (Gorrod et al., 2013). Proxy indicators that rely on expert judgement should be used with caution as assessor experience levels may result in varied opinions of the same site (Gordon et al., 2016). Indicators such as vegetation structure and grazing intensity are particularly vulnerable to observer bias as they are reliant on the timing of the assessment and the level of surveyor experience of the habitat. To reduce the chances of error, indicators should be clearly defined to reduce ambiguity in their interpretation and have raw data recorded, such as average vegetation height instead of visual grazing assessments. This also allows for the adjustment of the yearly scores, should the scorecard need to be recalibrated. Applying scorecards with a fixed species list across a range of landscapes poses the problem of regional variation in the plant community. A scorecard that is suitable for use in one region may not be as accurate when applied to another, even if the habitats are classified the same. Recalibration is an important feature to consider when testing scorecards, but it may carry risk of damaging landowner trust if executed midway through a project.

These scorecards are designed for rapid, single survey assessment and so have reduced the sampling strategies used in traditional biological sampling, such as quadrats, transects, and relevés, in favour of those that suit rapid assessment. This favours visual estimates of percentage cover of positive indicator species alongside presence/absence surveys. Whilst this is undoubtedly rapid and easily accomplished, it does not adequately address the reasons why traditional biological sampling methods were developed. For example, percentage estimates often require training to reduce user variation within a quadrat. However, there is no size limit set on the range of a scorecard. In our study, site sizes varied from 13 to 215 ha. Discerning an accurate assessment of percentage cover for bryophytes is likely to be an impossible task, particularly when the range of scoring categories is small, such as in the PMP (0–5%, 6–20%, 20–30%, > 30%). Without an adequate sampling strategy, the variation in site scores over time may be impossible to separate from natural variation or user variation (Magurran, 2021). This limits the ability to accurately assess the impacts of conservation efforts.

Scorecards need to remain simple enough to be flexible and practical, and so there is inevitably a balance to be struck between sampling effort and accuracy (Elmiger et al., 2023). The number of metrics chosen, and their weighting will define the clarity of the message the scorecard is conveying as measuring too many variables makes it hard to decipher the leading causes of change. This issue becomes apparent when scorecards are designed to be multi-habitat. Each additional habitat that is added to a scorecard’s range will lose a degree of accuracy for all covered habitats. Stewart and Jones (2020) tested the HHP and PMP peatland scorecards on blanket bogs in the Outer Hebrides and did not find them to be an accurate representation of ecological condition across the mosaic of habitats that comprise heathland landscapes. To address this, the authors developed a more general habitat condition scorecard covering various grasslands and heaths, rather than make separate habitat-specific scorecards (Stewart & Jones, 2020).

Addressing the issue of practicality and flexibility does not require a substantial overhaul of current methods or increasing the time spent in the field. Methods exist to incorporate Common Standards Monitoring into scorecards in Yorkshire, Wales, and Scotland (Stewart & Jones, 2020; POBAS., 2022). These cards require species to be present in a given number of stops, which helps to address the lack of frequency data in the positive indicator species variable and limits the influence of one patch overshadowing the condition of the whole site, providing that bias in stop placement is addressed. This method also incorporates a ranking system for positive indicator species, and scores are awarded based on the presence of high-ranking positive indicators and a selection of more commonly associated species. This helps elevate sites with rarer or more unique species assemblages from sites that have a large number of the more common indicators.

Scorecards rarely account for area or landscape-scale features despite their importance to the distribution of species such as birds, carabidae, and lepidoptera (Jeanneret et al., 2003; Merckx et al., 2009; Peach et al., 2001). This is in part due to the requirement for the indicators used in results-based AES to be within the control or influence of landowners (Ruas et al., 2021). However, assigning a single condition score over large, heterogenous landscapes such as an upland heath becomes a difficult task. A potential solution to this is to divide sites into land parcels, separated by condition or change of habitat, as is the practice in the PMP. However, opinion is split on this solution, as landowners may see low-scoring parcels as ‘sacrificial’ and discourage improvements (Gorman, unpublished data). Developing scorecards that are decoupled from an AES, and instead provide an ecological condition score to monitor restoration efforts, would not be bound by such restrictions.

Ultimately, there is a pressing need to increase our monitoring capabilities of Annex I habitats across the EU, yet priority areas included in results-based schemes are being monitored annually with project-specific methods producing data that is incompatible with wider monitoring. There is currently a missed opportunity to link these monitoring programs and produce valuable data to assist national monitoring efforts. The IBECA framework (Jakobsson et al., 2021) developed for forest and alpine ecosystems in Norway is an example of how a quantitative and cost-effective framework can be developed and we believe a similar approach may be viable with ecological scorecards.

Before seeking to adapt ecological scorecards to new purposes, we should consider the following questions:

1. What are we measuring and how well do the existing scorecard metrics represent it? Is there a clear scientific link between the metrics and the target?
2. To what extent can good sampling strategy be employed in the survey method whilst remaining a rapid assessment?
3. How closely does the scorecard represent ecological condition assessments for related protected habitats?
4. Does the scorecard need recalibration to reflect regional variation?

If we can adequately answer these questions to provide multi-user scorecards for Annex I habitats, we could increase our monitoring capabilities and potentially empower local communities to monitor their own environment and become engaged in restoration efforts. Failure to address these may lead to inappropriate monitoring strategies being used in future conservation projects, wasting valuable resources. This would be particularly important in regard to the extent of peatland restoration planned in Ireland. Quantifying restoration efforts in the rewetting of bogs would be an ideal role for a new scorecard, if they can be shown to accurately measure peatland recovery.

## Conclusion

Current Irish peatland and heathland scorecards provide an excellent communication tool between ecologists and land managers and have been instrumental in the widening success of results-based AES, but caution is needed before we assume true ecological condition based on their score, or their applicability for uses outside of these schemes. However, with some adaptions to their methodology, they could provide a powerful tool with which we could drastically increase ecological monitoring. In particular, we recommend that scorecards either choose to be habitat specific, or alternatively become more generalist, as one card is unlikely to be representative of large heterogenous landscapes. Finally, the recording of raw data alongside categorical scores would help increase the value of the project-specific data, as there is currently a lost opportunity to provide wider monitoring data whilst ecologists are in the field monitoring protected habitats .

## Appendix

See Table 3

**Table 3:** Site scores achieved by the selected scorecards. Average scores for each scorecard and the range of scores for each site are also shown. Site names have been removed as they are part of an ongoing agri-environmental scheme

Heath	BFF	HHP	PMP	RBAPS	Test LA	Range
1	7	4	6.5	6	6.8	3
2	7	8	8.5	7	8.5	1.5
3	7	8	8.5	5	8.8	3.8
4	7	4.5	7	3.5	7.2	3.7
5	3	1	− 0.5	− 1	4.8	5.8
6	1.5	− 3	− 1.3	0	2.3	5.3
7	7.5	7	7	6	8.7	2.7
8	5	3	2	0	6.4	6.4
9	7.5	7	7.5	6	7.2	1.5
Average score:	5.83	4.39	5.02	3.61	6.74	3.74

### Supplementary Information

Below is the link to the electronic supplementary material.

Supplementary file1 (DOCX 20.5 KB)

## Data Availability

No datasets were generated or analysed during the current study.

## Footnotes

1 (92/43/EEC Article 1e) The conservation status of a natural habitat will be taken as ‘favourable’ when: Its natural range and areas it covers within that range are stable or increasing, and. The specific structure and functions which are necessary for its long-term maintenance exist and are likely to continue to exist for the foreseeable future, and. The conservation status of its typical species is favourable as defined in (i).

## References

[CR1] Agri-Climate Rural Environment Scheme (ACRES). 2022. Available at https://www.gov.ie/en/service/f5a48-agri-climate-rural-environment-scheme-acres. Accessed 11 Aug 2023.

[CR2] Balmford, A., Gaston, K. J., Blyth, S., James, A., & Kapos, V. (2003). Global variation in terrestrial conservation costs, conservation benefits, and unmet conservation needs. *Proceedings of the National Academy of Sciences,**100*(3), 1046–1050. 10.1073/pnas.0236945100 10.1073/pnas.0236945100 PMC298723 12552123

[CR3] Batáry, P., Dicks, L. V., Kleijn, D., & Sutherland, W. J. (2015). The role of agri-environment schemes in conservation and environmental management. *Conservation Biology,**29*(4), 1006–1016. 10.1111/cobi.12536 25997591 10.1111/cobi.12536 PMC4529739

[CR4] Birds Directive 2009/147/EEC. Directive 2009/147/EEC of the European Parliament and the Council of 30 November 2009 on the conservation of wild birds

[CR5] Borges-Matos, C., Maron, M., & Metzger, J. P. (2023). A review of condition metrics used in biodiversity offsetting. *Environmental Management,**72*(4), 727–740. 10.1007/s00267-023-01858-1 37477675 10.1007/s00267-023-01858-1

[CR6] Brooks, R. P. (1997). Improving habitat suitability index models. *Wildlife Society Bulletin (1973-2006),**25*(1), 163–167.

[CR7] Brown, E. D., & Williams, B. K. (2016). Ecological integrity assessment as a metric of biodiversity: Are we measuring what we say we are? *Biodiversity and Conservation,**25*(6), 1011–1035. 10.1007/s10531-016-1111-0 10.1007/s10531-016-1111-0

[CR8] Bull, J. W., Suttle, K. B., Gordon, A., Singh, N. J., & Milner-Gulland, E. J. (2013). Biodiversity offsets in theory and practice. *Oryx,**47*(3), 369–380. 10.1017/S003060531200172X

[CR9] Burren Programme – Farming for Conservation. (2021). http://burrenprogramme.com/. Accessed 19 Oct 2022

[CR10] CAP23. (2023). *Climate Action *Plan* 2023*. Government of Ireland.

[CR11] Caughlan, L., & Oakley, K. L. (2001). Cost considerations for long-term ecological monitoring. *Ecological Indicators,**1*(2), 123–134. 10.1016/S1470-160X(01)00015-2 10.1016/S1470-160X(01)00015-2

[CR12] Concepción, E. D., Díaz, M., & Baquero, R. A. (2008). Effects of landscape complexity on the ecological effectiveness of agri-environment schemes. *Landscape Ecology,**23*(2), 135–148. 10.1007/s10980-007-9150-2 10.1007/s10980-007-9150-2

[CR13] Delbosc, P., Lagrange, I., Rozo, C., Bensettiti, F., Bouzillé, J.-B., Evans, D., Lalanne, A., Rapinel, S., & Bioret, F. (2021). Assessing the conservation status of coastal habitats under Article 17 of the EU Habitats Directive. *Biological Conservation,**254*, 108935. 10.1016/j.biocon.2020.108935 10.1016/j.biocon.2020.108935

[CR14] Díaz-Delgado, R., Hurford, C., & Lucas, R. (2017). Introducing the Book “*The Roles of Remote Sensing in Nature Conservation*”. In R. Díaz-Delgado, R. Lucas, & C. Hurford (Eds.), *The Roles of Remote Sensing in Nature Conservation: A Practical Guide and Case Studies* (pp. 3–10). Springer International Publishing. 10.1007/978-3-319-64332-8_1

[CR15] EEA. (2020). State* of nature in the EU — European Environment Agency* [Publication]. https://www.eea.europa.eu/publications/state-of-nature-in-the-eu-2020. Accessed 6 Jun 2023

[CR16] Ellwanger, G., Runge, S., Wagner, M., Ackermann, W., Neukirchen, M., Frederking, W., Müller, C., Ssymank, A., & Sukopp, U. (2018). Current status of habitat monitoring in the European Union according to Article 17 of the Habitats Directive, with an emphasis on habitat structure and functions and on Germany. *Nature Conservation,**29*, 57–78. 10.3897/natureconservation.29.27273 10.3897/natureconservation.29.27273

[CR17] Elmiger, B. N., Finger, R., Ghazoul, J., & Schaub, S. (2023). Biodiversity indicators for result-based agri-environmental schemes – Current state and future prospects. *Agricultural Systems,**204*, 103538. 10.1016/j.agsy.2022.103538 10.1016/j.agsy.2022.103538

[CR18] *Farm *Advisor* Resources | Freshwater Pearl Mussel Ireland | Pearl Mussel Project*. (2022). https://www.pearlmusselproject.ie/resources/farm-advisor-resources.html. Accessed 3 Oct 2022

[CR19] Fennessy, M. S., Jacobs, A. D., & Kentula, M. E. (2007). An evaluation of rapid methods for assessing the ecological condition of wetlands. *Wetlands*, *27*(3), Article 3. 10.1672/0277-5212(2007)27[543:AEORMF]2.0.CO;2

[CR20] Gordon, B., Rothrock, P. E., & Labus, P. (2016). Testing the use of best professional judgment to create biological benchmarks for habitat assessment of wetlands and oak savannas in northwestern Indiana. *Ecological Indicators,**60*, 410–419. 10.1016/j.ecolind.2015.07.014 10.1016/j.ecolind.2015.07.014

[CR21] Gorman, T., Kindermann, G., & Morley, T. (2019). A rapid assessment framework for Irish habitats: A case study of machair habitat quality. *Irish Geography*, *51*(2), Article 2. 10.2014/igj.v51i2.1372

[CR22] Gorrod, E. J., Bedward, M., Keith, D. A., & Ellis, M. V. (2013). Systematic underestimation resulting from measurement error in score-based ecological indices. *Biological Conservation,**157*, 266–276. 10.1016/j.biocon.2012.09.002 10.1016/j.biocon.2012.09.002

[CR23] Haase, P., Tonkin, J. D., Stoll, S., Burkhard, B., Frenzel, M., Geijzendorffer, I. R., Häuser, C., Klotz, S., Kühn, I., McDowell, W. H., Mirtl, M., Müller, F., Musche, M., Penner, J., Zacharias, S., & Schmeller, D. S. (2018). The next generation of site-based long-term ecological monitoring: Linking essential biodiversity variables and ecosystem integrity. *Science of the Total Environment,**613–614*, 1376–1384. 10.1016/j.scitotenv.2017.08.111 10.1016/j.scitotenv.2017.08.111 29898505

[CR24] Habitats Directive, 1992/43/EEC. Council Directive 92/43/EEC of 21 May 1992 on the conservation of natural habitats and of wild flora and fauna. *Official Journal of the European Union*

[CR25] Hen Harrier Project. (2021). *Hen Harrier Project—Resources*. Hen Harrier Project - Resources. Accessed 15^th^ May 2021. Available at http://www.henharrierproject.ie/resources.html

[CR26] Hodge, I., Hauck, J., & Bonn, A. (2015). The alignment of agricultural and nature conservation policies in the European Union. *Conservation Biology,**29*(4), 996–1005. 10.1111/cobi.12531 25998969 10.1111/cobi.12531

[CR27] Jakobsson, S., Evju, M., Framstad, E., Imbert, A., Lyngstad, A., Sickel, H., Sverdrup-Thygeson, A., Töpper, J. P., Vandvik, V., Velle, L. G., Aarrestad, P. A., & Nybø, S. (2021). Introducing the index-based ecological condition assessment framework (IBECA). *Ecological Indicators,**124*, 107252. 10.1016/j.ecolind.2020.107252 10.1016/j.ecolind.2020.107252

[CR28] Jeanneret, P., Schüpbach, B., & Luka, H. (2003). Quantifying the impact of landscape and habitat features on biodiversity in cultivated landscapes. *Agriculture, Ecosystems & Environment,**98*(1), 311–320. 10.1016/S0167-8809(03)00091-4 10.1016/S0167-8809(03)00091-4

[CR29] JNCC (2009) Common Standards Monitoring Guidance for Upland Habitats, Version July 2009, JNCC, Peterborough, ISSN 1743–8160.

[CR30] Kleijn, D., & Sutherland, W. J. (2003). How effective are European agri-environment schemes in conserving and promoting biodiversity? *Journal of Applied Ecology*, *40*(6), Article 6. 10.1111/j.1365-2664.2003.00868.x

[CR31] Lindenmayer, D. B., & Likens, G. E. (2011). Direct measurement versus surrogate indicator species for evaluating environmental change and biodiversity loss. *Ecosystems,**14*(1), 47–59. 10.1007/s10021-010-9394-6 10.1007/s10021-010-9394-6

[CR32] Lovett, G. M., Burns, D. A., Driscoll, C. T., Jenkins, J. C., Mitchell, M. J., Rustad, L., Shanley, J. B., Likens, G. E., & Haeuber, R. (2007). Who needs environmental monitoring? *Frontiers in Ecology and the Environment,**5*(5), 253–260. 10.1890/1540-9295(2007)5[253:WNEM]2.0.CO;2 10.1890/1540-9295(2007)5[253:WNEM]2.0.CO;2

[CR33] Magurran, A. E. (2021). Measuring biological diversity. *Current Biology,**31*(19), R1174–R1177. 10.1016/j.cub.2021.07.049 34637726 10.1016/j.cub.2021.07.049

[CR34] Medeiros, H. R., & Torezan, J. M. (2013). Evaluating the ecological integrity of Atlantic forest remnants by using rapid ecological assessment. *Environmental Monitoring and Assessment,**185*(5), 4373–4382. 10.1007/s10661-012-2875-7 22996823 10.1007/s10661-012-2875-7

[CR35] Merckx, T., Feber, R. E., Riordan, P., Townsend, M. C., Bourn, N. A. D., Parsons, M. S., & Macdonald, D. W. (2009). Optimizing the biodiversity gain from agri-environment schemes. *Agriculture, Ecosystems & Environment,**130*(3), 177–182. 10.1016/j.agee.2009.01.006 10.1016/j.agee.2009.01.006

[CR36] Milberg, P., Bergstedt, J., Fridman, J., Odell, G., & Westerberg, L. (2008). Observer bias and random variation in vegetation monitoring data. *Journal of Vegetation Science,**19*(5), 633–644. 10.3170/2008-8-18423 10.3170/2008-8-18423

[CR37] Moran, J., Byrne, D., Carlier, J., Dunford, B., Finn, J., Ó hUallacháin, D., & Sullivan, C. (2021). Management of high nature value farmland in the Republic of Ireland: 25 years evolving toward locally adapted results-orientated solutions and payments. *Ecology and Society*, *26*(1). 10.5751/ES-12180-260120

[CR38] Morrison, L. W. (2016). Observer error in vegetation surveys: A review. *Journal of Plant Ecology,**9*(4), 367–379. 10.1093/jpe/rtv077 10.1093/jpe/rtv077

[CR39] NPWS, 2019a. The Status of Protected EU Habitats and Species in Ireland. Overview Volume 1. Unpublished Report, National Parks and Wildlife Services. Department of Arts, Heritage and the Gaeltacht, Dublin, Ireland.

[CR40] NPWS, 2019b. The Status of Protected EU Habitats and Species in Ireland. Overview Volume 2. Unpublished Report, National Parks and Wildlife Services. Department of Arts, Heritage and the Gaeltacht, Dublin, Ireland.

[CR41] Oliver, I., Eldridge, D. J., Nadolny, C., & Martin, W. K. (2014). What do site condition multi-metrics tell us about species biodiversity? *Ecological Indicators,**38*, 262–271. 10.1016/j.ecolind.2013.11.018 10.1016/j.ecolind.2013.11.018

[CR42] Parkes, D., Newell, G., & Cheal, D. (2003). Assessing the quality of native vegetation: The ‘habitat hectares’ approach. *Ecological *Management* & Restoration*, *4*(s1), Article s1. 10.1046/j.1442-8903.4.s.4.x

[CR43] Pe’er, G., Birkenstock, M., Lakner, S., & Röder, N. (2021). The Common Agricultural Policy post-2020: Views and recommendations from scientists to improve performance for biodiversity. Volume 2 - Annexes (Working Paper 175-Volume 2). Thünen Working Paper. 10.3220/WP1620647428000

[CR44] Peach, W. J., Lovett, L. J., Wotton, S. R., & Jeffs, C. (2001). Countryside stewardship delivers cirl buntings (Emberiza cirlus) in Devon. *UK. Biological Conservation,**101*(3), 361–373. 10.1016/S0006-3207(01)00083-0 10.1016/S0006-3207(01)00083-0

[CR45] Pearl Mussel Project. (2021). *PMP Publications | Freshwater Pearl Mussel Ireland | Pearl Mussel *Project. PMP Publications. https://www.pearlmusselproject.ie/resources/publications.html. Accessed 3 Oct 2022

[CR46] Perrin, P.M., Barron, S.J., Roche, J.R. & O’Hanrahan, B. (2014). Guidelines for a national survey and conservation assessment of upland vegetation and habitats in Ireland. Version 2.0. Irish Wildlife Manuals, No. 79. National Parks and Wildlife Service, Department of Arts, Heritage and the Gaeltacht, Dublin, Ireland.

[CR47] POBAS, 2022. Piloting* an Outcome Based Approach in Scotland*. Available at: https://www.nature.scot/doc/piloting-outcomes-based-approach-scotland-pobas-project-phase-2-report. Accessed 15^th^ January 2024

[CR48] R Core Team. (2023). A language and environment for statistical computing. *R Foundation for Statistical Computing*, Vienna, Austria. Available at: https://www.R-project.org/. Accessed 10 May 2022

[CR49] RBAPSProject. (2021) Accessed 16th April 2022). *RBAPS* Project. https://rbaps.eu/

[CR50] Rotchés-Ribalta, R., Ruas, S., Ahmed, K. D., Gormally, M., Moran, J., Stout, J., White, B., & hUallacháin, D. O. (2021). Assessment of semi-natural habitats and landscape features on Irish farmland: New insights to inform EU Common Agricultural Policy implementation. *Ambio,**50*, 346–359. 10.1007/s13280-020-01344-6 32472434 10.1007/s13280-020-01344-6 PMC7782645

[CR51] Ruas, S., Rotchés-Ribalta, R., hUallacháin, D. Ó., Ahmed, K. D., Gormally, M., Stout, J. C., White, B., & Moran, J. (2021). Selecting appropriate plant indicator species for Result-Based Agri-Environment Payments schemes. *Ecological Indicators,**126*, 107679. 10.1016/j.ecolind.2021.107679 10.1016/j.ecolind.2021.107679

[CR52] Scott, W. A., & Hallam, C. J. (2003). Assessing species misidentification rates through quality assurance of vegetation monitoring. *Plant Ecology,**165*(1), 101–115. 10.1023/A:1021441331839 10.1023/A:1021441331839

[CR53] Siddig, A. A. H., Ellison, A. M., Ochs, A., Villar-Leeman, C., & Lau, M. K. (2016). How do ecologists select and use indicator species to monitor ecological change? Insights from 14 years of publication in ecological indicators. *Ecological Indicators,**60*, 223–230. 10.1016/j.ecolind.2015.06.036 10.1016/j.ecolind.2015.06.036

[CR54] Sullivan, C., & Moran, J. (2017). The development of a draft peatlands and heathland scorecard using the Results-Based Agri-environmental Pilot Scheme (RBAPS) scorecard approach. Available at https://rbaps.eu/documents/scorecards. Accessed 20^th^ February 2022

[CR55] Stewart, R. & Jones, G. (2020). Developing results-based approaches to supporting the management of common grazings - final report, vol. 1. *European Forum on Nature Conservation and Pastoralism*.

[CR56] Tubridy, M., Iremonger, S., Hickey, B., O’Hanrahan, B., & Tubridy, M. (2015). *Blackstairs habitat mapping and biodiversity audit 2015*. 63. Unpublished report.

[CR57] Uthes, S., & Matzdorf, B. (2013). Studies on agri-environmental measures: A survey of the literature. *Environmental Management,**51*(1), 251–266. 10.1007/s00267-012-9959-6 23086399 10.1007/s00267-012-9959-6

[CR58] Vanden Borre, J., Paelinckx, D., Mücher, C. A., Kooistra, L., Haest, B., De Blust, G., & Schmidt, A. M. (2011). Integrating remote sensing in Natura 2000 habitat monitoring: Prospects on the way forward. *Journal for Nature Conservation,**19*(2), 116–125. 10.1016/j.jnc.2010.07.003 10.1016/j.jnc.2010.07.003

[CR59] Vos, P., Meelis, E., & Ter Keurs, W. J. (2000). A framework for the design of ecological monitoring programs as a tool for environmental and nature management. *Environmental Monitoring and Assessment,**61*(3), 317–344. 10.1023/A:1006139412372 10.1023/A:1006139412372

