# Supplementary material for: Variation in ecological scorecards and their potential for wider use
Source: Environ Monit Assess. 2024 Jul 10;196(8):722. doi: 10.1007/s10661-024-12845-2 (PMC11236852; doi:10.1007/s10661-024-12845-2)
Supplement: Supplementary file 1 — Supplementary file1 (DOCX 20.5 KB) [file 10661_2024_12845_MOESM1_ESM.docx]

---

title: "Simulated_scorecard"

author: "Kevin Healy"

date: "2023-05-03"

output:

pdf_document: default

html_document: default

---

```{r setup, include=FALSE}

knitr::opts_chunk$set(echo = TRUE)

```

## Simulate score card

First we set all the possible scores each category can have

```{r ind_scores}

VegSwt <-c(0,5,10,15)

PISNowt <-c(0,10,15,20)

PISCovwt <- c(0,10,15,20)

NISNowt <- c(0)

NISCovwt <- c(0,-5,-10,-15,-20)

Grazwt <- c(-15,-10,0,10,15)

WaterImpwt <- c(0)

HydroIntwt <- c(0)

Drainwt <- c(-15,-10,-5,5,15)

Bryowt <- c(0,5,10)

Scrubwt <- c(5,-5,-10,-15)

Brackenwt <- c(0,-5,-10,-15)

Baresoilwt <- c(0,-5,-10,-15)

Burningwt <- c(0,-5,-15)

Disturbwt <- c(0,-5,-15)

Turbarywt <- c(0)

SuppFwt <- c(0)

```

Now we simulate 1000000 random scores where every score is equally likely.

```{r total score}

#create empty list

total_score <- vector()

sim_length <- 1000000

total_score <- sample(VegSwt, sim_length, replace = TRUE) +

sample(PISNowt, sim_length, replace = TRUE) +

sample(PISCovwt, sim_length, replace = TRUE) +

sample(NISNowt, sim_length, replace = TRUE) +

sample(NISCovwt, sim_length, replace = TRUE) +

sample(Grazwt, sim_length, replace = TRUE) +

sample(WaterImpwt, sim_length, replace = TRUE) +

sample(HydroIntwt, sim_length, replace = TRUE) +

sample(Drainwt, sim_length, replace = TRUE) +

sample(Bryowt, sim_length, replace = TRUE) +

sample(Scrubwt, sim_length, replace = TRUE) +

sample(Brackenwt, sim_length, replace = TRUE) +

sample(Baresoilwt, sim_length, replace = TRUE) +

sample(Burningwt, sim_length, replace = TRUE) +

sample(Disturbwt, sim_length, replace = TRUE) +

sample(Turbarywt, sim_length, replace = TRUE) +

sample(SuppFwt, sim_length, replace = TRUE)

```

Lets plot out that distribution

```{r plot rand score}

hist_rand_score <- hist(total_score, breaks = 50)

plot(hist_rand_score)

```

#Biased scores

Lets now simulate the scores based on existing data (based on intial scores)

```{r set bias score}

VegSprob <- c(0.25,0.3,0.3,0.15)

PISNoprob <- c(0.15,0.25, 0.3, 0.3)

PISCovprob <- c(.15,0.25,0.3,0.3)

NISNoprob <- c(1)

NISCovprob <- c(0.3,0.3,0.2,0.15,0.05)

Grazprob <- c(0.25,0.3,0.2,0.15,0.1)

WaterImpprob <- c(1)

HydroIntprob <- c(1)

Drainprob <- c(0.15,0.25,0.2,0.25,0.15)

Bryoprob <- c(0.1,0.4,0.5)

Scrubprob <- c(0.3,0.35,0.25,0.1)

Brackenprob <- c(0.15,0.25,0.3,0.3)

Baresoilprob <- c(0.2,0.25,0.3,0.25)

Burningprob <- c(0.2,0.4,0.4)

Disturbprob <- c(0.35,0.35,0.3)

Turbaryprob <- c(1)

SuppFprob <- c(1)

```

```{r total biased score}

#create empty list

total_bias_score <- vector()

total_bias_score <- sample(VegSwt, sim_length, prob = VegSprob, replace = TRUE) +

sample(PISNowt, sim_length, prob = PISNoprob, replace = TRUE) +

sample(PISCovwt, sim_length, prob = PISCovprob, replace = TRUE) +

sample(NISNowt, sim_length, prob = NISNoprob, replace = TRUE) +

sample(NISCovwt, sim_length, prob = NISCovprob, replace = TRUE) +

sample(Grazwt, sim_length, prob = Grazprob, replace = TRUE) +

sample(WaterImpwt, sim_length, prob = WaterImpprob, replace = TRUE) +

sample(HydroIntwt, sim_length, prob = HydroIntprob, replace = TRUE) +

sample(Drainwt, sim_length, prob = Drainprob, replace = TRUE) +

sample(Bryowt, sim_length, prob = Bryoprob, replace = TRUE) +

sample(Scrubwt, sim_length, prob = Scrubprob, replace = TRUE) +

sample(Brackenwt, sim_length, prob = Brackenprob, replace = TRUE) +

sample(Baresoilwt, sim_length, prob = Baresoilprob, replace = TRUE) +

sample(Burningwt, sim_length, prob = Burningprob, replace = TRUE) +

sample(Disturbwt, sim_length, prob = Disturbprob, replace = TRUE) +

sample(Turbarywt, sim_length, prob = Turbaryprob, replace = TRUE) +

sample(SuppFwt, sim_length, prob = SuppFprob, replace = TRUE)

```

```{r plot bias score}

hist_bias_score <- hist(total_bias_score, breaks = 50)

plot(hist_bias_score)

```

#Correlated scores

Now lets now simulate the scores assuming that certain individual scores are

correlated. In this case lets assume that if

```{r correlate probs}

#create empty list

total_corr_score <- vector()

VegSwt_corr <-sample(VegSwt, sim_length, prob = VegSprob, replace = TRUE)

PISNowt_corr <- vector()

#if we want to correlate PISNowt with VegSwt_corr we can set the propoabilty of

#drawing scaores in another catagory depending on what we drew in the other.

#Its a bit messy but we can do it using a loop and if else statements

#Below is an example where we correlate VegSwt and PISNowt

for(i in 1:sim_length){

if(VegSwt_corr[i] == 0)

{PISNowt_corr[i] <- sample(PISNowt, 1, prob = c(0.90,0.05,0.03,0.02))}

else {if(VegSwt_corr[i] == 5)

{PISNowt_corr[i] <- sample(PISNowt, 1, prob = c(0.04,0.90,0.04,0.01))}

else {if(VegSwt_corr[i] == 10)

{PISNowt_corr[i] <- sample(PISNowt, 1, prob = c(0.01,0.04,0.90,0.04))}

else {if(VegSwt_corr[i] == 15)

{PISNowt_corr[i] <- sample(PISNowt, 1, prob = c(0.02,0.03,0.05,0.90))}

}

}

}

}

#lets also correlate Baresoilwt and Brackenwt

Baresoilwt_corr <-sample(Baresoilwt, sim_length, prob = Baresoilprob, replace = TRUE)

Brackenwt_corr <- vector()

for(i in 1:sim_length){

if(Baresoilwt_corr[i] == 0)

{Brackenwt_corr[i] <- sample(Brackenwt, 1, prob = c(0.01,0.03,0.06,0.9))}

else {if(Baresoilwt_corr[i] == -5)

{Brackenwt_corr[i] <- sample(Brackenwt, 1, prob = c(0.01,0.02,0.9,0.02))}

else {if(Baresoilwt_corr[i] == -10)

{Brackenwt_corr[i] <- sample(Brackenwt, 1, prob = c(0.02,0.9,0.02,0.01))}

else {if(Baresoilwt_corr[i] == -15)

{Brackenwt_corr[i] <- sample(Brackenwt, 1, prob = c(0.9,0.06,0.03,0.01))}

}

}

}

}

```

Once we create the correlated scores we can now just calculate the total

like before.

```{r correlate score}

total_corr_score <- vector()

total_corr_score <- VegSwt_corr +

PISNowt_corr +

sample(PISCovwt, sim_length, prob = PISCovprob, replace = TRUE) +

sample(NISNowt, sim_length, prob = NISNoprob, replace = TRUE) +

sample(NISCovwt, sim_length, prob = NISCovprob, replace = TRUE) +

sample(Grazwt, sim_length, prob = Grazprob, replace = TRUE) +

sample(WaterImpwt, sim_length, prob = WaterImpprob, replace = TRUE) +

sample(HydroIntwt, sim_length, prob = HydroIntprob, replace = TRUE) +

sample(Drainwt, sim_length, prob = Drainprob, replace = TRUE) +

sample(Bryowt, sim_length, prob = Bryoprob, replace = TRUE) +

sample(Scrubwt, sim_length, prob = Scrubprob, replace = TRUE) +

Brackenwt_corr +

Baresoilwt_corr +

sample(Burningwt, sim_length, prob = Burningprob, replace = TRUE) +

sample(Disturbwt, sim_length, prob = Disturbprob, replace = TRUE) +

sample(Turbarywt, sim_length, prob = Turbaryprob, replace = TRUE) +

sample(SuppFwt, sim_length, prob = SuppFprob, replace = TRUE)

```

Now lets plot the biased histogram

```{r corr plot}

host_corr <- hist(total_corr_score, breaks = 50)

plot(host_corr)

```

Now lets plot them all together

```{r full combined plots}

plot(host_corr,

col = rgb(69,53,73,max = 255, alpha = 50,))

title(main="Random, Biased, and Correlated score Models")

plot(hist_bias_score ,add = TRUE,

col = rgb(215,89,51,max = 255, alpha = 50))

title(main="Random, Biased, and Correlated score Models")

plot(hist_rand_score,add = TRUE,

col = rgb(249,194,111,max = 255, alpha = 50))

title(main="Random, Biased, and Correlated score Models")

```

#Here I will run the random score generator for the HHP, PMP, and BFF

#HHP indicator score allocation

VegSwt <-c(0,20,40)

PISNowt <-c(0)

PISCovwt <- c(0)

NISNowt <- c(0,-10)

NISCovwt <- c(10,-10,-20)

Grazwt <- c(0)

WaterImpwt <- c(-20,0,10)

HydroIntwt <- c(0)

Drainwt <- c(-20,0,10)

Bryowt <- c(0)

Scrubwt <- c(-10,0,10)

Brackenwt <- c(-30,-20,-10,0)

Baresoilwt <- c(-20,0,10)

Burningwt <- c(-20,0,10)

Disturbwt <- c(-20,-10,0)

Turbarywt <- c(-30,-20,0)

SuppFwt <- c(-20,-10,0)

total_scoreHHP <- vector()

sim_length <- 1000000

total_scoreHHP <- sample(VegSwt, sim_length, replace = TRUE) +

sample(PISNowt, sim_length, replace = TRUE) +

sample(PISCovwt, sim_length, replace = TRUE) +

sample(NISNowt, sim_length, replace = TRUE) +

sample(NISCovwt, sim_length, replace = TRUE) +

sample(Grazwt, sim_length, replace = TRUE) +

sample(WaterImpwt, sim_length, replace = TRUE) +

sample(HydroIntwt, sim_length, replace = TRUE) +

sample(Drainwt, sim_length, replace = TRUE) +

sample(Bryowt, sim_length, replace = TRUE) +

sample(Scrubwt, sim_length, replace = TRUE) +

sample(Brackenwt, sim_length, replace = TRUE) +

sample(Baresoilwt, sim_length, replace = TRUE) +

sample(Burningwt, sim_length, replace = TRUE) +

sample(Disturbwt, sim_length, replace = TRUE) +

sample(Turbarywt, sim_length, replace = TRUE) +

sample(SuppFwt, sim_length, replace = TRUE)

hist_rand_scoreHHP <- hist(total_scoreHHP, breaks = 50)

plot(hist_rand_scoreHHP)

#PMP indicator score allocation

VegSwt <-c(-15,-10,0,15)

PISNowt <-c(0,2,5,10)

PISCovwt <- c(0)

NISNowt <- c(0,-10)

NISCovwt <- c(10,-5,-10,-15)

Grazwt <- c(0)

WaterImpwt <- c(0)

HydroIntwt <- c(0,5,15)

Drainwt <- c(-30,-15,0,5,15)

Bryowt <- c(0,10,15,20)

Scrubwt <- c(0)

Brackenwt <- c(0)

Baresoilwt <- c(-20,-10,0,10)

Burningwt <- c(-15,-5,5)

Disturbwt <- c(-30,-20,-10,0)

Turbarywt <- c(-30,-15,-5,0)

SuppFwt <- c(-15,-5,0)

total_scorePMP <- vector()

sim_length <- 1000000

total_scorePMP <- sample(VegSwt, sim_length, replace = TRUE) +

sample(PISNowt, sim_length, replace = TRUE) +

sample(PISCovwt, sim_length, replace = TRUE) +

sample(NISNowt, sim_length, replace = TRUE) +

sample(NISCovwt, sim_length, replace = TRUE) +

sample(Grazwt, sim_length, replace = TRUE) +

sample(WaterImpwt, sim_length, replace = TRUE) +

sample(HydroIntwt, sim_length, replace = TRUE) +

sample(Drainwt, sim_length, replace = TRUE) +

sample(Bryowt, sim_length, replace = TRUE) +

sample(Scrubwt, sim_length, replace = TRUE) +

sample(Brackenwt, sim_length, replace = TRUE) +

sample(Baresoilwt, sim_length, replace = TRUE) +

sample(Burningwt, sim_length, replace = TRUE) +

sample(Disturbwt, sim_length, replace = TRUE) +

sample(Turbarywt, sim_length, replace = TRUE) +

sample(SuppFwt, sim_length, replace = TRUE)

hist_rand_scorePMP <- hist(total_scorePMP, breaks = 50)

plot(hist_rand_scorePMP)

#BFF indicator score allocation

VegSwt <-c(0,5,10,15)

PISNowt <-c(0,10,15,20)

PISCovwt <- c(0,10,15,20)

NISNowt <- c(0)

NISCovwt <- c(0,-5,-10,-15,-20)

Grazwt <- c(-15,-10,0,10,15)

WaterImpwt <- c(0)

HydroIntwt <- c(0)

Drainwt <- c(-15,-10,-5,5,15)

Bryowt <- c(0,5,10)

Scrubwt <- c(5,-5,-10,-15)

Brackenwt <- c(0,-5,-10,-15)

Baresoilwt <- c(0,-5,-10,-15)

Burningwt <- c(0,-5,-15)

Disturbwt <- c(0,-5,-15)

Turbarywt <- c(0)

SuppFwt <- c(0)

total_scoreBFF <- vector()

sim_length <- 1000000

total_scoreBFF <- sample(VegSwt, sim_length, replace = TRUE) +

sample(PISNowt, sim_length, replace = TRUE) +

sample(PISCovwt, sim_length, replace = TRUE) +

sample(NISNowt, sim_length, replace = TRUE) +

sample(NISCovwt, sim_length, replace = TRUE) +

sample(Grazwt, sim_length, replace = TRUE) +

sample(WaterImpwt, sim_length, replace = TRUE) +

sample(HydroIntwt, sim_length, replace = TRUE) +

sample(Drainwt, sim_length, replace = TRUE) +

sample(Bryowt, sim_length, replace = TRUE) +

sample(Scrubwt, sim_length, replace = TRUE) +

sample(Brackenwt, sim_length, replace = TRUE) +

sample(Baresoilwt, sim_length, replace = TRUE) +

sample(Burningwt, sim_length, replace = TRUE) +

sample(Disturbwt, sim_length, replace = TRUE) +

sample(Turbarywt, sim_length, replace = TRUE) +

sample(SuppFwt, sim_length, replace = TRUE)

hist_rand_scoreBFF <- hist(total_scoreBFF, breaks = 50)

plot(hist_rand_scoreBFF)

#Combined plots of BFF, HHP, PMP

plot(hist_rand_scoreHHP, col = rgb(215,89,51,max = 255, alpha = 50), main ="Score Comparison model of the HHP, PMP, and BFF Scorecards", xlab='Score', ylim=c(0,100000))

plot(hist_rand_scoreBFF, col = rgb(69,53,73,max = 255, alpha = 50), add=TRUE)

plot(hist_rand_scorePMP,add = TRUE, col = rgb(249,194,111,max = 255, alpha = 50))

BFFmean <- mean(total_scoreBFF)

HHPmean <-mean(total_scoreHHP)

PMPmean <-mean(total_scorePMP)

print(BFFmean)

print(HHPmean)

print(PMPmean)
